# The Reciprocal Influence of Body and Mind Considered; as It Affects the Great Questions of Education, Phrenology, Materialism, Moral Advancement, and Responsibility,—Man's Free Agency, the Theory of Life, the Peculiarities of Mental Property, Mental Diseases, the Agency of Mind upon the Body, of Physical Temperament upon the Manifestations of Mind and upon the Expression of Religious Feelings

**Published:** 1843-04

**Authors:** 


### Art. X.

*The Reciprocal Influence of Body and Mind considered; as it affects the great questions of Education, Phrenology, Materialism, Moral Advancement, and Responsibility,—Man's free agency, the theory of life, the peculiarities of mental property, mental diseases, the agency of mind upon the body, of physical temperament upon the manifestations of mind and upon the expression of religious feelings.* By W. Newnham, Esq. m.r.s l.—*London*, 1842. 8vo, pp. 628.

Mr. Newnham is known as an author in two capacities: He has written several works of a strictly religious kind, one of which has had an extensive circulation; as well as some scientific pamphlets, (such as an Essay on the Disorders of Literary Men, and a Report on the Progress of Surgery,) indicating his knowledge of the modern literature of his profession, his literary industry, and his own practical acquirements. In the present volume he has indulged his tastes by uniting his favorite topics, as it embraces subjects which require in their investigation the knowledge both of religious and medical science. It was the opinion of a metaphysician, whose views have had an extensive influence on the thinkers of the present day, that mental philosophy could never be thoroughly investigated, except by one who was versed in physiology and pathology, as well as in the actions of the mind itself abstracted from matter; a view which a medical man who has witnessed the manifold and strange influences of the body on the mind, and has considered the workings of his own mind, will admit the truth of without hesitation. And when the difficulty of the investigation, its complexity, extent, and practical importance are considered, any attempt to throw light upon its darkness by one who possesses, in a measure, both these qualifications, must be looked at with pleasure. Mr. Newnham's previous writings show that the study of mental phenomena has been a favorite one for many years. He is evidently a serious-minded man, sincerely and earnestly convinced of the reality of religious influences, and of their paramount importance; and a large practice for a long series of years must have regularly supplied him with objects of reflection, with facts, with matters, both to form his views, theories, or speculations, and to test their correctness.

Fearlessly to acknowledge the immense influence of matter on the outward manifestations of the mind can best be done by one who is deeply convinced that mind and matter are not identical; that mind is a great power, an agent, using matter as its medium. But still more necessary is it that a writer who, in the present time, boldly marks out the influence of physical temperament upon the expression of religious feeling, should himself be the subject of and therefore the unaffected believer in religious influences. In a scientific point of view this must be necessary. Unless a man has felt himself the influence of religion on his own mind and body, he is unable accurately and fully to understand its influence upon others. For a writer who addresses himself to general readers, especially to those of a religious cast, the necessity of this is obvious. Unless they believe in his sympathy with themselves in spiritual matters, and in his full recognition of feelings and of motives they themselves possess, they will not readily believe him when he explains to them that deep despondency, or, still more unlikely, extravagant hope and joy, are feelings which depend not so much on the condition of their soul, as on an irritable condition of their splanchnic nerves, or an accelerated circulation through the brain. We would illustrate this by an anecdote. A clergyman's wife, a strong-minded, serious person, was applied to, in the absence of her husband, by a woman for religious consolation in mental despondency of an exaggerated kind. The morbid body was as evident as the morbid mind. The lady gave her some opening pills, and told her to call again. Under similar circumstances, a medical man who had suggested the same remedy might have been considered but little better than one of the wicked.

It is true, to a certain extent, that religion does not come under our jurisdiction as medical reviewers; but all branches of knowledge are so connected together as to touch each other at some points, although in others their distinction is most obvious. The colours of the sky at sunset may be most various; the deep blue of the zenith may be in the strongest contrast with the deep orange of the horizon; but trace them as they join, and you cannot mark where one begins and the other ends, so harmoniously do they melt into each other: and so it is with all knowledge. Man divides it for his convenient study, and nothing is clearer than his divisions if regarded at those distances where the opposition and the contrast are greatest; but who can draw the line at others? It is not our business to discuss the meaning of religious terms, or weigh the merits of peculiar doctrines; we have nothing to do with the thirty-nine articles or with No. XC., but religion, regarded as an influence, or even merely as a subject which powerfully engages the thoughts and feelings, and therefore acts upon the brain and nervous system, comes within our daily observation, and demands our scrutiny. How many thousands are there in Great Britain at the present moment who make religious reading their single intellectual exercise, and for whom religious observances stand in the place of all diversions, excitements, and amusements? They are surely occupied with something which intensely engages their attention, deeply interests their feelings, and of the reality of which they are entirely convinced—something, which in many instances is believed to be the cause of the profoundest melancholy, or the completest mental happiness; of the most hopeless insanity of individuals; of the wildest vagaries or of the most sober, serious, matter-of-fact earnestness of whole communities; of all the forms which under the names of enthusiasm or of fanaticism have supplied so many pages of the history of mankind. Who, then, that studies the human mind can omit the contemplation of these phenomena? And if the mind does act upon the body, and the body reciprocally upon the mind, no investigator of this mutual influence can neglect this branch of examination.

The wards of a madhouse most fearfully illustrate these strong influences when exaggerated and distorted by disease. That bloated, red-nosed, middle-aged mechanic, who roars at you as you look into his cell, pouring out a torrent of blasphemous and obscene slang, thinks himself "God;" and those two weak feeble women, who are cowering together in close, quiet conversation, believe themselves to be Christ and the Holy Spirit; whilst the moping melancholics, wandering alone, are the victims of the most morbid religious despair. And, in a less degree, the medical practitioner meets in his daily rounds with cases illustrating the same influences,—sometimes morbid, producing undue fear and depression which counteract all physical means; sometimes healthful, producing a condition of mind in disease eminently conducive to recovery, or, where the complaint is incurable, entirely reconciling the individual to his lot: and, however willing he may be to acknowledge the power of mind over matter, he cannot but be struck with the constant influence of the body upon such states of mind, and the expressions of feelings regarded perhaps by the subject of them and his friends as exclusively spiritual. This being the case, we feel we are not stepping out of our province by giving Mr. Newnham's views on this branch of the medical philosophy of the mind and of its organ.

A considerable portion of this volume is taken up with the subjects of life, materialism, phrenology, man's free agency and responsibility, the differences between his mind and those of brutes, &c. They are discussed by one who is not disposed indolently to shirk any question bearing upon his principal subject, but willing to place each side of it in every light before his own mind, and to decide for himself its value. His endeavour obviously is to be fair, honest, and true; and he is always charitable. With these branches of the subject our space will not allow us to grapple, as our wide differences with the author on many points would entail long discussions; we shall confine ourselves to the main subject, the reciprocal influence of the mind and body, extracting, analysing, and compressing what appears either new or else well stated, as the result of Mr. Newnham's own observation and reflection.

The contents of the whole volume (a very thick one) have evidently been written at various times when the particular subject was fresh in the writer's mind, and often at long intervals; and the author's occupation, an extensive country practice, is one of all others least calculated to encourage concentrated or continuous thought. This explains a certain prolixity and diffuseness, and a deficiency of clear order and arrangement which makes our task somewhat difficult; but we must also explain that we shall be obliged often to curtail most extensively the author's course of reasoning, which is generally too long to be given entire. We think it also necessary to state that while dissenting from many of Mr. Newnham's physiological and psychological views, we shall not stop to discuss the point with him, as the belief entertained respecting the precise nature of such phenomena does not vitiate the arguments, much less destroy the importance of the facts adduced in illustration of the main investigation—the reciprocal influence of body and mind. The meaning of the terms cannot be mistaken, although entirely different views may be entertained of the nature of the things to which the terms are applied.

*Education, moral and physical.* The chief object of Mr. Newnham's work is to direct attention to the influence of the temperament of the body on the mind, in health and in disease. Education (he says) may modify, soften, direct, and improve the mind; but it cannot change the physical temperament, which always gives a tinge to the thoughts, feelings, and actions of the individuals. The kind and degree of mental power are dependent upon physical constitution. Differences in intellect and in attainments depend on the greater or less perfection of the brain, and its adaptation for peculiar pursuits. The immaterial principle presides over the brain, through which its operations are manifested. The brain is the servant, the mind is fitted to govern and to direct it, so that man is a free agent. "By his *will* he can control, suspend, or encourage any one of his peculiarities." He is consequently responsible for all he does, says, or thinks. Hence the necessity of education, especially when young, "the free agency of the man depending upon the education of the child; for if this be not what it ought to be, he will become the slave of his passions and propensities, and he will lose his intellectual and his spiritual freedom." This education, consequently, is of two sorts; the education of the body and of the mind.

"A first claim to attention is presented by all which relates to the health of the body, and of those several organs and functions which in their influence, immediate or remote, exert a generic or specific agency upon the organ of mind: and a second, and not inferior claim, is to be found for all those processes, through which the dominion of intellect may be enlarged, the evil passions and propensities may be controlled, and the mind may be prepared for that moral and religious training which is to complete the perfection of man as a rational and accountable being." (p. 28.)

To continue our analysis. What is true of other organs is true of the brain—that to secure healthy function the organ must be *exercised*: that lengthened repose is fatal to its *tone*: and that excessive exertion, or irritative action, will produce diminished power, or feebleness. The capacity of the brain for exertion is progressive: it cannot be stationary. It is a mistake to suppose that the brain will suffer from judicious exercise. The danger is in "fitful exertion," great exercise after long inaction. Gradual employment is to be pursued, so as not to induce excessive or irritative action, which may be occasioned by long-continued exertion giving rise to exhaustion, and followed by irritability, which state may be mistaken for *power*, but which will eventually terminate in feebleness. The same morbid state may be brought about by stimuli, or by the lavish excitement of the feelings, and of emotion. The brain and the stomach are in close sympathetic connexion, and if the stomach is overtaxed the brain is weakened and oppressed, and eventually its powers are impaired, and permanent disorganization follows: hence the necessity of controlling the stomach.

Mr. Newnham's psychical physiology is of the most complex kind: he believes that life is a principle added to organization, and that man has a spiritual principle added to life, which distinguishes him from brutes, but which is "characterized, modified, and sometimes perverted," by the brain through which it acts.

*Effect of the passions.* The influence of the passions on animal life is discussed, and the following passage is given as the explanation of the mode in which painful conditions of the mind impair the general health:

"A painful impression is made, is perceived by the brain, and communicated to the mind, which becomes preoccupied by it, and makes, in consequence, large demands on the cerebral agency; then nervous influence which is daily produced in order to support the bodily actions, and the mental manifestations, is too exclusively taken up by the latter—not so much, perhaps, for the purpose of manifestation, as for the infinitely more consuming office of bearing up under, or concealing the constant misery of a wounded spirit. Concentrated and hidden suffering is that which makes the largest demands upon the brain. Now, the result of this is, that a certain quantity only being daily produced, and an undue proportion being given to spiritual suffering, enough is not left to maintain the functions of organic life in all their integrity: the supply of nervous energy to the respective organs is irregular, or insufficient, or perverted; and disorder of the animal functions is the consequence." (p. 258.)

"A certain amount of nervous energy is necessary to the function of digestion; cut off this supply entirely, and digestion is impossible; let it be insufficient, and digestion is only partially accomplished; let it be perverted, and a depraved form of digestion occurs; irritation and uneasiness of the stomach are produced, with acidity and a long train of horrors; these produce a reflex influence upon the brain, shown first in unwonted sleepiness and inaptitude for intellectual pursuits, followed closely by dreams, and uneasy and unrefreshing sleep; next morning the patient gets up listless and feeble, and the bodily as well as the intellectual functions are disturbed; headach and irritability are the result; nervous energy is wasted upon trifles, or worse than trifles; the hour of dinner recurs; there is less power of digestion than the day before; and this goes on oftentimes in a rapidly-augmenting ratio, till the stomach becomes the seat of chronic inflammation, and ulceration, or other morbid action, which, by slow degrees, conducts the patient to the tomb, the victim of his own actions, and frequently the miserable and the voluntary suicide of his own better feelings and principles." (p. 261.)

Where the stomach itself escapes, the food is imperfectly digested, and the process of sanguification is consequently impaired; nutrition is impossible; the patient grows thin; the brain is insufficiently supplied with blood, and nervous power is impaired and unequally distributed, producing various local irritations, distress of the head, less aptitude for intellectual pursuits, a greater prevalence of the peculiar temperament with diminished power of the will, till the weakest organ fails, and local being superadded to general disorder, the system is at length worn out.

The influence of "the interior life upon the body" is shown by many little well-known circumstances.

"Witness the suffusion of the countenance in blushing; the shrunk features and pale goose-skin produced by alarm; the chattering of the teeth under fear; the increase of various secretions from mental emotion, as of the tears in sorrow, or the bile in anger; the palpitation of the heart, under almost every sudden emotion, the short and quickened breathing of expectation, the oppressed and stifled respiration of intense and harrowing emotion, the arrested and almost imperceptible action of breathless anxiety and expectancy; the influence upon the muscles of expression of the countenance, alternately lighted up with joy, or worn with anxiety or suffering, and the thousand varied emotions which they are capable of expressing; the plump portliness of the man at ease, and the extreme thinness of the victim of deep disappointment, or of any long-continued devouring passion; so that to be dried by grief—to be devoured by remorse—to be consumed by sorrow—are not only common expressions, but literal representations of actual bodily conditions." (p. 263.)

It has been urged as an argument against materialism that the mind becomes more mature as the body decays. Mr. N. very justly combats this, and shows that man's moral nature may improve, he may use more self-control, he may have more equanimity, benevolence, and love to others; but that the power of his intellect diminishes as the brain, like the rest of the body, becomes more feeble.

That the brain partakes of the general decay, is shown by literary labour becoming irksome; the power of application, and the love of pursuit, diminished; the perception slow, the imagination extinct, the memory of the immediate past lost, judgment infirm and vacillating. The sight is often the first to give way, the hearing becomes less acute, the touch and smelling obtuse, and all cease to convey slight impressions or accurate notices to the intelligent principle within. The feebleness of the voice, and of the power of locomotion, equally show the diminution of nervous power as age creeps on.

Even the *spiritual principle* (Mr. N. says) is liable to disorder! Any disturbance of this spirit will irritate the brain, and render it unfit for the full and perfect performance of its function; and every little bodily ailment will prevent the brain from being the willing servant of the spirit within.

*Conscience.* The influence of physical disorder on the conscience is thus described:

"Conscience is a faculty which insanity often distorts, and which the minor disturbances of the body very frequently pervert, by producing a degree of fearfulness and hesitation, which render man uncertain in his opinions, changeful in his judgment, and vacillating in action; he becomes doubtful upon trifles; he magnifies their importance; he wishes to do right, but cannot discover what is right; when he thinks he has attained to a just judgment, he is turned aside by some veriest straw in the scale of moral action; he becomes the slave of superstitious observances; he is always desirous of propitiating the goodwill of his neighbours, and of deprecating the wrath of the Almighty; yet he seeks to accomplish this object, not by the firmness and fearlessness of right, but by seeking to please others. The frequent failure thus produced will occasion remorse, and this again will give rise to a very unfavorable and depressing influence upon the powers of life.

*Reflection.* "Intimately connected with the power of conscience," says Mr. N. "is the habit of *reflection*......But there are morbid states of this process: man may become *too reflective* a character; his associations with exterior life may be broken up; he may be lost in his own thoughtfulness; become deaf to the impressions of exterior life, from which his knowledge is to be derived; and while occupied with his reflections he may allow the time for action to pass by. The contemplative character, and, with a still deeper shade, the pensive, are morbid states of reflection, and, as such, ought to be avoided." (p. 342)

*Memory.* Mr. Newnham next considers memory. He has several times noticed a good memory become feeble and worthless from morbid action in the brain. Sometimes the injury has been permanent, in others it has been slowly recovered when the diseased action has ceased. In such cases the attention is first impaired, which will account for the impaired recollection of immediate or recent impressions; but the loss extends to circumstances which had formerly made a lively impression, and had been remembered up to the time of the invasion of the physical disorder. This is an important symptom, leading to the early detection of cerebral disease; and the earliest indications should be attended to. Sometimes the individual feels he has not the power of directing and fixing his attention to any subject which involves consecutive reasoning. Even in common reading, he finds he reads and reads again, without being one jot the wiser, and this happens not from the common cause—of the thoughts directed elsewhere.

*Absence of mind* may be a symptom of disease of the brain; it is at all times a formidable symptom, not that it may not exist without disease, but it is a frequent early symptom of cerebral mischief. In another condition, impressions on the senses make so little impression, or such incorrect ones, as to stimulate the brain erroneously. The patient is in a kind of dream; involuntary trains of thought enjoy the control of the mind. An aggravation of these states occurs when no impressions are received at all.

"Let, then, the first symptoms of unwonted forgetfulness—of unusual listlessness—of unaccustomed indisposition to exertion—of diminished energy—of shrinking from application—of retirement from duty—of omission of details—of perversion of thought, of reasoning, or plans—let any one of these symptoms be early discovered, and zealously watched, for there is no time to lose; the brain must be saved, or death or insanity may follow." (p. 348.)

It is hardly necessary to remind our readers that the importance to be attached to these symptoms of mental debility depends on any of them being foreign to the patient's ordinary condition of mind,—otherwise, how many of our patients would afford a very gloomy prognosis! The following is an important practical hint:

"This state is one which will not generally bear depletion; it does not consist in too much arterial action, nor in venous congestion, but in torpor of the nervous fibre, which will commonly be augmented by depleting measures. Nothing is more mischievous than to mistake sensorial loss of energy for vascular loss of balance; and the distinction being easy the mistake should never be made." (p. 349.)

*Imagination.* The faculty of imagination has a large influence upon disease. When morbid, the individual becomes superstitious and fanatical; he attends to dreams, and to omens; he listens to voices, and receives impulses which have no foundation in reason or religion; he is deceived by hallucinations, and ultimately becomes monomaniacal. As imagination acts so largely on the hopes and fears, it operates considerably on almost every bodily disorder, and its "judicious management, on the contrary, will often be the means of prolonging or of destroying life. Hence the importance of sedulously watching its agency, and especially of sedulously guarding it from eccentric action." (p. 351.)

*Hysteria.* Mr. Newnham adduces hysteria, to explain how greatly the actions of the body and mind are perverted by slight and temporary irritation of the brain. In such persons the nervous temperament largely predominates, and a slight mental emotion will, as is well known, "suspend the action of the heart, disorder respiration, produce muscular spasms, alter secretions, cause violent pain where there is no cause for pain, suspend the action of the brain, producing unconsciousness, or pervert its action, as in involuntary crying and laughing, &c." (p. 406); and all this shall pass, and in a few hours, or minutes, the patient is well again, and only feels exhaustion.

*Fainting* illustrates the same point: the brain fails, from failure of the heart to supply it with blood, and this often from the slight causes acting on the nervous system; emotion, a disagreeable sight, a peculiar odour, a painful reminiscence.

"A disposition to frequent fainting is not uncommon, though it may not amount to a complete suspension of action; and this is a very painful state, as it tends most entirely to unfit its subject for thought, and other intelligent manifestation; and yet the patient remains feelingly alive to the change." (p. 408.)

*Mental decay.* The gradual decay of the brain occurs in different individuals at very varying periods of life,—precociously, in those whose nervous system has been originally feeble, or in whom it has been impaired by intense thought, or anxiety, or domestic misery, or by politics, or by wine, spirits, or opium. But even in the most favorable circumstances, a gradual decay takes place. The individual has at first less power to command attention; he has to read and reread his author to obtain the meaning, from finding "*he cannot give his mind to it.*" He cannot concentrate his thoughts on any subject; hence his views are imperfect, and consequently he is prejudiced. The consciousness of this loss of power makes him peevish and irritable, impatient of contradiction, and disposed to substitute petulance for argument. At last, power over the brain is lost. The symptoms of decay vary according to the causes, which Mr. N. arranges under six heads.

1. In exhaustion of the brain there is commonly distress about the head, often severe headach with quick pulse, "the pulse of effort," with increased vascular action about the head; wakefulness; painful consciousness that the brain requires absolute rest, but with heavy broken sleep, dreaming, and a thousand waking fancies; study is onerous, and increases the symptoms.

2. Congestion of the brain is marked by torpor, and diminished sensibility; dull, heavy weight about the head, and often giddiness; pulse slow, full, labouring; general weakness, or prostration of strength; great sleepiness by day and night; gloominess, perceptions dull. The brain is benefited by some exertion, even intellectual, but especially by bodily exercise.

3. Loss of nervous power generally from over-action of the brain, is not always dependent on congestion, though sometimes connected with it; and as at first there is not much visible disease, the patient "very generally gets blamed for something wrong in his *morale*."

"Thus indolence of manner and conduct is often ascribed to the want of mental industry; he lies in bed when he ought to be up; he will be found indolently lounging upon his sofa, when he ought to be studying; the letter-writing of to-day will be postponed till to-morrow, as will the ride, the walk, the errand of charity, the business; there is an absence of interest in all he says and does; he appears to want the proper amount of social feeling, and he is inattentive to the wants, the desires, the entreaties of those around him. By-and-bye, cerebral malady stands out in full force; the cause of all this alteration of character becomes acknowledged; the disease leads to much physical infirmity, and to the gradual extinction of life." (p. 417.)

Mr. N. calls this condition of brain "*sensorial torpor*."

4. "Precisely opposite to this state is the form of *irritable brain*. This seems dependent upon some preceding unwonted action, during which the fibre is rendered too susceptible to impression, and the brain has not power enough to control that irritability. He feels excessively the merest trifles—he is quick, irritable, irascible, prone to take offence, and always on the *qui vive* for something to awaken this irritability." (p. 417.)

5. The state of the brain may depend on the alteration in the general mass of blood, without any decreased action in the brain itself, but merely that slow or increased vascular action which is compatible with ordinary health. If the vascular action is excited, the ideas flow rapidly, imagination is more fertile, perception more acute, the feelings intense, the judgment more accurate, and if this state is preserved within the line of health, good is produced. But it is a dangerous excitement, often followed by a sluggish action of the veins, from their having been over-filled, and gradually losing their power of getting rid of the blood in due time for the next quantity. In time, the arterial action becomes accommodated to the slow venous movement, and the mind becomes torpid,—drowsiness, general languor, indifference, and distaste for thought, and a feeble, hesitating, ever-changing judgment.

6. Lastly, a form of nervous irritation, accompanied by an apprehension of impending evil. The patient is "exceedingly nervous, absurdly fearful, as it might be called; too diffident to act, too distrustful to be firm; too wavering to be relied upon, and with a prevailing expectation of future evil, that evil varying from the common ills of life to the highest evil, exclusion from the hope of forgiveness, of happiness, of heaven."

All these states are liable to spectral illusions, to hallucinations, and to insanity.

Mr. N. alludes to a form of cerebral disorder, "when some distant irritation ceases all at once, and is converted into cerebral malady assuming the form of hallucination. If the original malady can be reproduced, the secondary evil will probably be relieved; but when this cannot be the case, it remains oftentimes permanently." In all hallucinations the brain "has escaped the province of reason," arguments are unavailing, the only remedy is to treat the morbid action in the brain.

*Insanity*. Mr. Newnham next passes to insanity, and remarks on the disadvantages of calling it a mental disease, as contradistinguished from bodily disorder, and very acutely shows that, making allowance for the organ affected, it differs in nothing from other diseases.

"There are the same period of incubation—the same premonitory symptoms of disorder—the same transient and slight derangement of function—the same march of fully-formed disease—the same advance and decline, marked, as in other maladies, not by constant progression, but by occasional advances and retrocessions—the one being greater than the other, according as the malady is loosening its hold, or narrowing its grasp upon the system, and always being marked by this one feature—that in the former case, the entire ground gained is never wholly lost; and in the latter, that the entire ground lost is never wholly regained; exacerbation and remission, called, in the case of insanity, paroxysm and lucid interval, are always to be found. Add to these points of similarity, that its terminations are the same; these being in restoration to health, which leaves the patient just as before the invasion of disorder; or by some other form of malady which seems to prove critical of the more important disorder; or by some alteration of function which leaves the original integrity more or less impaired; or by some change of structure, marked by increasing disturbance; or by the substitution of disease and such organic changes as are incompatible with restoration to health; or by complete loss of power, as in idiocy or atrophy; or by the gradual and complete extinction of life." (pp. 429-30.)

The treatment is the same, to discover and remove the cause—"subduing inordinate, or rousing languid vascular action," soothing irritation by giving sympathy, consolation, hope, and confidence; removing perverted trains of reason, and replacing them by correct ones. The necessity of patience is insisted upon. The strength of the morbid convictions depends on the patient's dwelling on an idea so constantly, that it becomes fixed, and the patient a monomaniac; and, for its cure, line upon line, precept upon precept, must be "judiciously employed, keeping in view the fact, that great changes are not to be effected in the nervous system except by time, and a frequent repetition of the means employed."

The boundary between actions arising from physical infirmity and from moral guilt, is defined with difficulty.

"Until we possess a better *test*," says Mr. N. charitably, "we may take this as our criterion.... Whatsoever is willingly, determinately, considerately, and deliberately done, must, if it be *wrong*, be placed to the score of *moral wrong*: and that wrong which occurs without the support of the will, independently of it, and even in opposition to its general bearing, may be allowed to partake of the nature of physical infirmity." (p. 433.)

In a healthy mind and brain the will has supreme power, commanding and changing the course of the thoughts. In insanity this power is lost.

"Hence arise uncontrolled thoughts; images over which the judgment has no influence; imaginations without foundation; impulses not to be accounted for; apparently sound reasoning upon false premises; constant change of purpose; action without definite object; disproportioned will, sometimes earnest over trifles, and almost nothing over objects of primary importance.... Hence arises the great analogy between insanity and dreaming." (p. 434.)

*Religious insanity.* Mr. N. treats at some length of the influence of religion on insanity. He combats the notion that religion, properly so called, can produce insanity. Religion, as "the best preserver from the influence of the animal passions—the best regulator of the affections, and the surest guide through the difficulties of life, affords the best security for the tempest-tossed brain, to those who obey its precepts." But in a brain *predisposed to insanity*, in which a strong emotion will overturn its integrity, religion may be said to produce insanity in two ways. 1. By its natural influence, as setting before a man the highest hopes and greatest fears, involving future happiness or misery. And when an individual predisposed to insanity, and who has for many years been careless about these things, earnestly turns his attention to them, it is not surprising that the subject engrosses him, even exclusively. This exclusive attention keeps up one set of impressions, and the reason is overturned. 2. By its unnatural influence, when the predisposed individual takes up false notions of an implacable God ; " when he contemplates himself as predestined to misery, and when he loses himself in the mysteries of election," an exclusive and depressing impression is produced, and is followed by one of the most fearful forms of insanity, and religion thus works like the passion of love, especially when unrequited ; like too much business to the merchant ; like ambition, or political trials ; the brain is morbidly impressed in all, the impression becomes exclusive, and the man insane. This pseudo-religious insanity may be divided into the hopeful and the desponding. In the former state, the maniac is full of his own self-importance, and of some extraordinary commission which he is to accomplish. In the second, he is without hope, and can receive no comfort, and this is often caused by false views of religious belief: substituting for that cheerfulness with which a right view of religion invests its believers, " a mysticism which makes it fearful." (p. 458.)

Temperament has a considerable influence over these states. The sanguine temperament inclines to hope, the melancholic to despair. In illustrating the effect of the mind upon the body, Mr. Newnham gives the following anecdote which occurred within his own circle :

" Three brothers entered into some most successful speculations, and realized very large profits ; but they continued the speculation ; one bargain more ! and now the tide had turned ; the article they had bought had been carried up by speculation to an unnatural height, and they could no longer sell except at a loss ; they still hoped for a favorable reaction, but depression was the order of the day ; to save their credit they must sell ; they could only do so at a ruinous sacrifice ; they could not meet their responsibilities ; next morning one had died by his own hands ; another was seized by apoplexy, and lived only a few hours ; the third died in a few months from ulceration of the stomach. Insanity had been rife in the family." (p. 472.)

Mr. Newnham discusses those cures, such as Prince Hohenloe's, which seem to be effected by the entire faith of the patient in the miraculous powers of another:

" Their experiments were never made upon a deaf and dumb person, upon a case of blindness, produced by disorganization of the eye, from spinal deformity, from destruction of the bodies of the vertebræ, upon the loss of a limb, or upon a disorganized joint. The cure was limited upon that class of chronic diseases which depends for its characteristic intensity upon a peculiar laxity of the nervous fibre ; a fact of primary importance, since it shows the influence of mind upon the bodily functions and structure in the cure of disease." (p. 481.)

In these cases in general, the effect was palliative only; it was " curative in those instances in which nature, or antecedent treatment had already effected a cure, and excitement alone was necessary to give energy to the weakened organs." The influence of mind (Mr. Newnham says,) can effect no permanent cure of any organic structural alteration.

*Announcement of expected death to patients.* In practice there is a subject of difficulty, delicacy, and importance which it is necessary that the medical attendant should have thoughtfully considered before he is called upon to act; and that is as to acquainting the patient with his danger. Mr. Newnham discusses this like one who has duly weighed an important question, and who has often put his thoughts into action. We refer those readers who wish to see this point practically discussed by a religious man to the volume itself: those who are of a similar disposition of mind, (and to those chiefly such observations are of service,) will find many special directions and hints for their guidance. We must confine ourselves to a short summary of the argument. It is the first duty of the medical attendant to preserve life. In this purpose confidence of the patient in his physician and in the remedies employed are of great service; so are hope and cheerfulness. On the other hand, a patient under such circumstances must not be deceived. The question is therefore as to the mode of communicating the intelligence. A hasty or brusque manner of revealing danger might at once kill the patient. The rule is "to speak the truth yet cheer the sufferer." In dangerous acute diseases, where the powers are sinking, to acquaint the patient with his danger would increase it without any compensating good. The patient is too much occupied with disease to think at all, or to think correctly. It is to chronic diseases that the question applies; in such there are many opportunities to lead a patient to the consideration of the uncertainty of life, to the frequent fatal issue of disease, the necessity of preparation, and the wisdom of making provision for the worst, even whilst expecting what is better, (see p. 496, et seq.) Such subjects should be introduced with delicacy and at proper opportunities; their injudicious management is very mischievous. If the patient inquires as to the probable issue of his disease, the best mode is to review candidly and honestly the usual progress of such a complaint, and the peculiarities of his individual case, and whilst giving all the hope which such peculiarities admit of, yet concluding that where there is uncertainty the safe course is to act for the best, but to be fully prepared for the worst; the course of a prudent man in the common evils of life.

*Temperament.* Mr. Newnham next considers the influence of temperament upon the mind.

1. *The sanguineous temperament*, marked by florid complexion, blue eyes, fair skin, light and often red hair, animated countenance, active and easily-excited circulation, firm and elastic muscles. Its disorders are accompanied by high action and power, so that it will bear exhausting remedies better than any other. The vis medicatrix naturæ is marked. The character of mind is similar, active and powerful, impressions received are lively, memory good, rich and lively imagination, fondness for society, benevolence and ardour; on the other hand it has its mental evils: irascible, easily provoked, disposition volatile, so that deeply-expressed sorrow is speedily followed by calm self-complacency, not persevering, fondness for novelties, friendships and hatreds of short duration. The cerebral diseases are of the same character, violence rather than depression, mania not hypochondriasis; in all there is always a certain degree of joyousness, self-content, and self-esteem.

2. *The bilious or choleric temperament*, marked by dark hair, eyes, and complexion, dry skin, strong muscular fibres not disposed to as much exertion as the sanguineous, but capable of longer effort; much mental power. The best literary characters are found in this temperament, from its constancy and perseverance. There is less quickness of mind, but greater solidity and depth; more attention to books than to persons; not the same fervour of affection, but greater constancy and greater self-denial to do good to the object; less agreeable, but more to be relied on; cautiousness and firmness. It is liable to bilious headachs, arising most frequently from the head, with which the stomach sympathises; subject to venous congestion of organs, especially of the head rather than high arterial action; it will not bear very active remedies; more liable to chronic disorder, "inasmuch as venous congestion takes place slowly, at first without exciting much notice or general disturbance, and thus the way is prepared for alteration of structure." Its mental peculiarities may become morbid from excess; its unchangeableness, prejudice; its consciousness, bigotry; its firmness, obstinacy; its caution, suspicion; its reserve, (a concentration into self,) unamiable; its courage, temerity; its fondness for reading may take off the man from the duties of action to the pleasures of abstraction; too great a disposition to find fault with the world. In its insanity there is a disposition to gloom; to look at the dark side of everything; to be weary of life; to believe that you are the sport of evil spirits and forsaken of God; and to self-destruction. This tendency to suicide in those of a peculiar temperament is not brought forward (says Mr. Newnham) to prove that one who commits suicide must be insane. The case is widely different from that brought on by moral despair, by acts and conduct of his own; when a man has lived a life of self-gratification, or of immorality, and when with a ruined fortune and a worn-out body he sees nothing but misery in the world, and futurity a blank; or when a man has lived a life of thoughtless extravagance, beggared himself and his family, and sees inevitable ruin before him; or when after diligent prosperity in business he is reduced by an unexpected reverse, and has no high principles to support him; or when the victim of seduction cannot bear the exposure of her shame—"in all these cases suicide is not insanity." It is a wilful error.

We give this argument, but still feel the difficulty of its application. In many such cases there may have been a disposition to insanity, which these causes have suddenly brought into activity; and therefore the act of suicide may be pronounced an act of insanity, without any justification of the previous causes which led to it, for then the individual is of course morally responsible; but the jury or the witness have to inquire into the sanity of mind at the moment of committing the act; and where there have been any previous symptoms of insanity, or any suspicion of an hereditary tendency to that disease, however culpable in a moral point of view may have been the individual's conduct, and however it may have tended to encourage the development of cerebral disease, we think the jury are justified in leaning to that construction of the Act which is the most merciful.

3. *The lymphatic temperament*, marked by a prevalence of the white fluids and of general feebleness; a pale and thick skin; great softness of muscle; soft light hair; soft, languid, light eyes; thick lips, which give the appearance of the mouth being half closed; and feebleness of character as well as of structure. It is liable to consumption and all forms of scrofula; and in all its disorders feebleness and inability to support action are characteristic, and when morbid action has commenced there is little chance of arresting it. Its mental characteristic is action without power to support it; as exemplified in precocious children. If called on for much exertion, the mind wears out the body and itself. So it is with the affections and passions of this temperament; a certain vehemence which cannot be sustained. A greater proportion of fools, idiots, and those with feeble minds among the insane belong to this class.

4. *The nervous temperament* is marked by its great susceptibility of impression and mobility; this restlessness keeps the individual thin; there is great changefulness of countenance; sudden animation, but often an expression of languor very distinctive. The influence of emotion may be sudden, and appear to be deep, but the feeling is quickly superseded by a new one, to be replaced by a third. Its diseases have a similar character, as the protean forms of hysteria indicate.

*Temper.* In alluding to the influence of the internal condition of the abdominal viscera on the mind, Mr. Newnham makes an acute remark on the *temper* which is worth remembering. Every one is sensible how much his mind varies with the stage or condition of his digestion, and that ill temper, moodiness, and irritability often proceed from the same causes. Such manifestations of ill temper, however, are usually kept as *home* disagreeables, the same man abroad being all smiles and kindness; hence it is evident that the will is successful in resisting their agency; and if the mind is capable of overcoming their organic suggestions, it is bound to do so at all times.

*Changes of opinion* are next discussed. The natural and healthful are of course the result of increased knowledge and wisdom; but there are others which depend on conditions of the brain, independent of this increase of wisdom. By morbid changes Mr. Newnham intends those which without adequate cause are at variance with the individual's former self; a sudden perversion of character, which throws an air of strangeness "over the whole mind; a perversity of thought and feeling, which needlessly gives undue prominence to certain points; an inaccessibility to reason and conviction," all at variance with common sense, and opposed to his previous views—a state bordering on insanity.

*Temperament and religion.* We next come to *the influence of temperament on the expression of religious feeling.* Religion consists in the service of the intellect as well as of the feelings; but there are two kinds of religionists, who seem to think that but one of these faculties is alone employed. The one whose religious feeling consists in a species of quietism, the belief of being a passive agent in the hands of Providence, having every event arranged and every feeling prompted by a spiritual influence, and of having nothing to do but to wait; and this character is alone found in the peculiar form of melancholic temperament. The opposite error is the religion of the head only, consisting in a love of contention for doctrines; bustling and overweening outward activity, especially in public; much talking without feeling, &c.; and this is found in one form of the choleric temperament. Not that such peculiarities may not arise from mental causes, but that a religionist, who has a strongly marked melancholic temperament, will exhibit such traces of its influence. Religion itself is not altered, but "the expression of religious feeling is characterized in no small degree by physical temperament." This assertion is illustrated by a sketch of the different effects produced by religious impressions on two individuals of opposite temperament. The individual of sanguineous temperament, when first he begins to think seriously on religion, is disposed to hope and to be joyful; the melancholic to despair from his past conduct; the former proclaims openly his change, the latter distrusts himself and conceals it, though equally sincere. The former becomes foremost "in all the bustling activity of charity," the latter, with no less sincerity, is less ostentatiously useful. In sickness, the former, whose character depended so much on health and on the integrity of his nervous system, becomes irritable and repining from his suspension of his activity. It is easier to do than to suffer; occupation is favorable to strength, and gives energy and confidence; but take away this prop, and patient endurance is exceedingly difficult. This is a physical condition, and there are few who have not experienced the benefit of walking or riding when wearied, worried, or perplexed. On the contrary, the person of melancholic temperament, who in health had less appearance of religion, now exhibits its real influence. Always accustomed to struggle against constitutional melancholy, he will not be depressed, he is patient, and submits from a conviction that all is for the best. The same distinction will appear at the approach of death. Supposing that both are dying of the same disease, the man of sanguineous temperament is full of hope and confidence; he of melancholic temperament has many fears and occasional doubts, although unshaken in his religious faith. Both have been influenced by the same principle, although their actual feelings, and more especially the expression of those feelings, were dissimilar.

On the same principles is explained the zeal and enthusiasm of one class, and the quiet steady pursuit of religion of another.

> "Constitutional tendency, and physical temperament will often account for that which perhaps might be called wordliness by one class, which class would in the views of the former be considered as remarkable for cant. Hence the former should not be regarded as irreligious, nor the latter as hypocritical."

*Prayer*. Some very good persons are distressed at their inability at times to pray. But this may depend on the body, as is shown in the same individual under a change of physical circumstances. "Only let sickness assail him, especially that which distresses the head: or even great bodily fatigue; and now an oppressive languor creeps upon the mind, blunts the feelings, distracts attention, perverts perception and destroys that gift of eloquent combination which before might have charmed us, &c." The same observation applies to the power of fixing the attention; in some persons, slight causes disturb the attention, and it will be found that they are equally liable to disturbances in the animal functions, owing to great irritability of their nerves. In such, a trifling bodily ailment interferes with their brain, and serious thought becomes oppressive. After long illness convalescence in such a patient is often attended with peevishness, and impatience from a feeling that he is inadequate to the performance of the duties he most desires, and cannot shake off his weakness, but must wait for a return of bodily strength. There is an opposite condition of the nervous system, where impressions do not easily produce morbid irritation; protracted suffering does not disturb in a great degree the nervous system, and the mind seems more independent of the body, and not influenced to the same extent by fatigue or exhaustion. This depends not on the constitution of the mind, but on the soundness of the brain. It is not the weak, but the strong organ of the body. Moral causes may still further strengthen this constitutional gift. Firm judgment, decision, prompt and persevering action, inflexible justice, and above all religion will do much towards establishing this original bestowment; or on the contrary their absence may render it worthless. The power of abstraction from self and surrounding objects, and of fixing the mind on things unseen is in great measure the result of these two causes; a healthy brain improved by mental discipline.

The occasional existence of vigour of mind and increased thoughts just before death has been brought forward as evidence of the immateriality of the soul; but (as Mr. Newnham says,) if this is evidence, the opposite condition would prove its materiality, and this latter state is by far the most frequent. The truth is that such manifestation depends on the health of the brain which may not be affected by the fatal disease, but such instances are rare; the general rule is "that the manifestations of mind are rendered feeble, or obscure, inefficient or perverted, exactly in proportion as its organ may have been subjected to the influence of disease." Many look to a death-bed as a test of the individual's principles. But Mr. Newnham's observation, grounded not on theory, but upon witnessing the death of many, is that "many a feeble Christian expires in trembling doubt, whilst many a careless sinner is remarkable for his calm, or rather his thoughtless dismissal; many a self-righteous pharisee leaves the world with exulting recollections of his own good works." So often is this the case, "that the nature of the malady, and the kind of temperament being given, it will be easy to predicate the peculiarities of the death-bed." Friends should not therefore rest their hopes or fears on the "precarious phenomena of dissolutions, characterized by physical temperament, encompassed by infirmity, and modified by a great diversity of organic irritation," but rather upon the previous life.

*Death.* The phenomena of death depend on the organ which dies first. If the brain dies first the manifestations of mind are generally obscured and perverted, but there is an occasional exception in cases where just before death, there is a "sudden lightening up," and a display of mental power the more striking from the previous condition. The fact is thus explained by our author;

"These phenomena are referrible to a physiological law that just before the giving way of any organ of the body, there is communicated to it and to the nervous system at large, a temporary stimulus, which gives to the patient a sensation as if the individual failing viscus were actually in a state of high health and vigour. This law may be easily exemplified; and indeed it is well known to all persons who have a weak digestion; under some circumstances, there will be a feeling of comfort and elasticity diffused through the system, and, as it would seem, irradiating from the very organ in fault, the stomach, which to the uninitiated patient speaks a decided improvement of health and strength; whereas, in five minutes afterwards, a consciousness of sinking and debility, and all the other phenomena of indigestion, will proclaim that this organ was only under the influence of temporary excitation, from the actual commencement of that morbid action which was to terminate in failing power." (p. 609.)

Such is a brief analysis of Mr. Newnham's views on a subject most worthy of attention. To the volume itself we must refer those who are so far interested in it as to wish to see it more fully discussed. Should one of our readers be disposed to believe that Mr. Newnham has over-rated the influence of the body on the mind in the exhibition of religious feelings, and to an extent likely to be injurious to a cause which both think of the greatest importance, we would beg of him to read the book itself, as its author has fully guarded his views from any such misapprehension, and our space has prevented our using many of his arguments. This last chapter (the tenth) we consider the most original and valuable, embracing the result of much actual experience on circumstances upon which a medical practitioner (whatever may be his own personal views or opinions,) must often have to converse, to decide, to afford consolation under the most painful circumstances, none of which he can do adequately if he has not previously made them the matter of consideration. We need hardly observe that much which we have quoted should be regarded as mere statements, the truth of which must be tested by comparison with actual facts falling beneath each individual's observation. But although it may not comprehend the whole truth, we believe there is much truth in it, and very much which is worthy of consideration. It is impossible to read Mr. Newnham's work without the conviction that he has bestowed very much thought on a large number of important subjects; and that he has kept these subjects for a long time before him, turning them over and looking at them from various points, patiently and perseveringly. In a scientific point of view we may regret that he has not aimed at concentration and compression, and that he has rather endeavoured to make his meaning intelligible by amplification, than by well weighing the exact meaning of each word; but still we would wish to keep in view, and to make allowance for his object, which is obviously that very difficult one, to write a book on an abstruse subject which general readers might understand, and scientific men profit by. This has been accomplished in the same department of knowledge, the philosophy of the mind, by a physician, second to no living man in the diligent and fruitful observation of disease, although in his advancing years he seems, as a writer, to have forsaken matter for mind, and certainly for general advantage. But Dr. Abercombie has gained the ear of the educated evidently by such studied brevity, as was consistent with clearness in the statement of truths in their nature somewhat obscure; a brevity infinitely more laborious than copious illustration and amplification, and only attained to by him who has that rare self-command, "the power of rejecting his own thoughts," a qualification (according to one whose prose was as lucid, and as concise as his poetry,) essential to a good writer. But Mr. Newnham can say with Madame de Sevigné, "chacun a son stile; le mien comme vous voyez, n'est pas laconique;" and if by such a style a larger number of readers are induced to think on a very important subject which has never before occupied their attention, and if what is still better, they become imbued with the charity and general benevolence of feeling towards others whom they see going astray or at least not walking in the same apparent path, which this volume inculcates, and gives reasons for, much good will be effected. Few will rise from its perusal without being sensible that on some points their views have become more clear, and that new trains of thought have been suggested to them; and no one can close it without feeling much respect both for the principles and the abilities of the author.

